# Cannabis-induced Acute Coronary Syndrome: A Coincidence or Not?

**DOI:** 10.7759/cureus.5696

**Published:** 2019-09-19

**Authors:** Eric Landa, Erika Vigandt, Alexander Andreev, Yury Malyshev, Sonu Sahni

**Affiliations:** 1 Internal Medicine, Ross University School of Medicine, Bridgetown, BRB; 2 Internal Medicine, Brookdale University Hospital Medical Center, New York, USA; 3 Cardiology, Maimonides Medical Center, New York, USA

**Keywords:** acute coronary syndrome, marijuana, myocardial infarction, st depression, kounis syndrome

## Abstract

Marijuana, derived from the *Cannabis sativa* plant, is the most commonly abused illicit drug in the United States. Now, more than ever, due to changing regulations, marijuana is more readily available and is known to be habitually used by millions. The neuropsychiatric effects of marijuana are well-known which include chronic fatigue syndrome and polyphagia. However, marijuana is also known to exert cardiac effects, such as tachycardia, hypotension, and hypertension. Marijuana has also been described in association with atrial fibrillation, ventricular tachycardia, and cardiac arrest. However, acute coronary syndromes, such as myocardial infarction in the setting of marijuana use, is rare. Herein, we present the case of a non-ST-elevation myocardial infarction (NSTEMI) in the setting of marijuana use in a 42-year-old African American male with no significant past medical history who presented with chest pain at rest one hour after smoking marijuana.

## Introduction

It is estimated that greater than 65 million Americans (31% of the United States (US) population aged 12 and older) currently or have participated in the use of marijuana [1-2]. Cardiovascular effects of marijuana on the heart have been documented; it can cause an increase in the heart rate that can range anywhere from 20% - 100% after smoking with the onset occurring within 10 - 30 minutes [3]. Other effects include a reduction in blood pressure, orthostatic hypotension, and cardiac arrhythmias (including atrial fibrillation, atrial flutter, and ventricular tachycardia) [3]. Different studies have demonstrated the relationship between marijuana and its chronotropic effects, as well as electrical disturbances [3-4]. Few of them speak to its role in myocardial ischemia (MI). Herein, we present the case of a marijuana-induced non-ST-elevation myocardial infarction (NSTEMI).

## Case presentation

A 42-year-old male with no significant past medical history presented to the emergency room at Brookdale University Hospital Medical Center with a chief complaint of chest pain. The patient reported that he was lying in bed watching television when he suddenly started feeling intense substernal chest pain. He described the pain as if someone was kicking him in the chest, rating the pain at 8/10 in severity. The pain was non-radiating, not reproducible, and without any alleviating or aggravating factors. It was associated with diaphoresis and one episode of non-bilious, non-bloody vomiting. The patient reported that he was smoking marijuana just one hour prior to the onset of the chest pain. His social history was significant for smoking marijuana six to seven times a day for the past 20 years, he was a social alcohol user, and denied any other illicit substance abuse, including cocaine.

**Figure 1:**
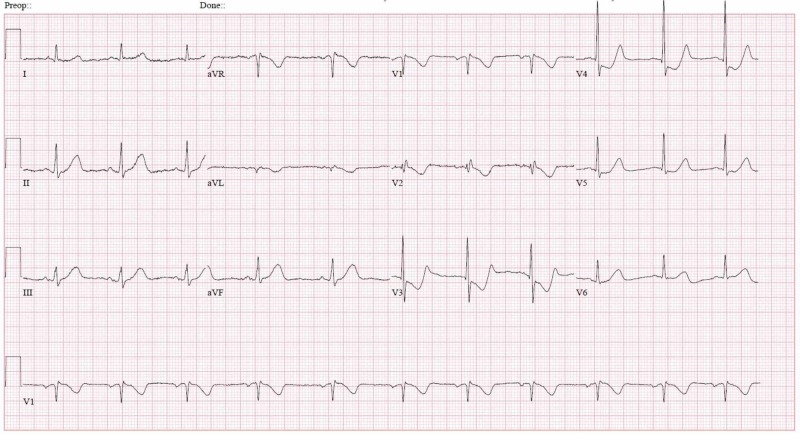
Electrocardiogram showing ST depression in V3 and V4 suggesting a non-ST-elevation myocardial infarction (NSTEMI)

Initial vital signs were within normal limits with blood pressure (BP) at 116/81 mmHg, an oral temperature of 36.2°C (97.2°F), a pulse of 61 beats/min, a respiratory rate of 18 breaths/min, and oxygen saturation of 97%. An electrocardiogram (EKG) revealed a significant ST depression in V3 and V4 which is shown in Figure 1.

**Figure 2:**
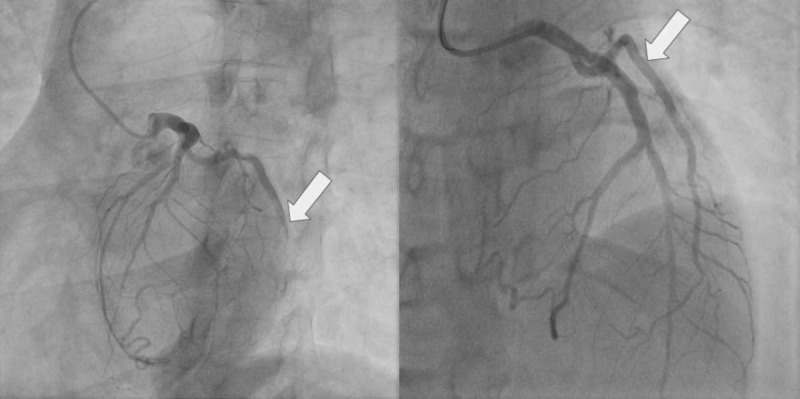
Complete occlusion of the first obtuse marginal artery (OM1) before percutaneous coronary intervention (PCI) (left) and after PCI (right)

Initial troponin was found to be mildly elevated at 0.044 ng/mL by laboratory standards but was clinically negative for myocardial infarction. A urine toxicology screen was positive for cannabinoids only. However, based on the EKG findings and symptomatology, the patient was treated as non-ST-elevation myocardial infarction (NSTEMI), and the percutaneous coronary intervention (PCI) team was activated. He was administered 325 mg of aspirin and 180 mg of ticagrelor and taken for PCI. He was found to have 100% stenosis (Thrombolysis in Myocardial Infarction (TIMI) flow 0) of the first obtuse marginal (OM) artery and the right coronary artery. An EluNIR™ (Cordis, Santa Clara, CA) 2.5 x 24 mm drug-eluting stent (ridaforolimus) was placed in the first OM as it was thought to be the culprit lesion causing the symptoms. Pre and post-PCI catheterization images are shown in Figure 2.

**Figure 3:**
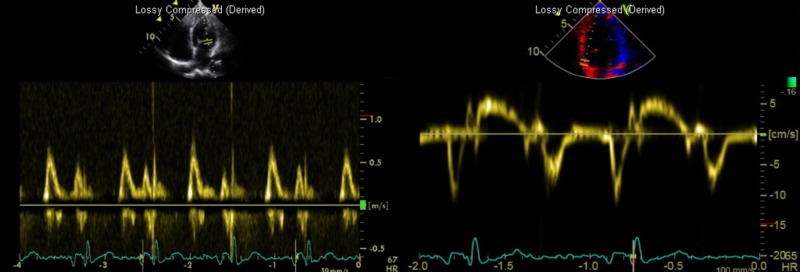
Echocardiogram imaging displaying mitral inflow Doppler (left) showing a normal E/A ratio and deceleration time. Tissue Doppler (right) shows an elevated e/e’ ratio indicative of Grade II (pseudonormal) diastolic dysfunction.

The patient reported that his chest pain was relieved after the procedure. EKG showed complete resolution of the ST depressions after PCI. Troponin was measured again post-procedure and found to be 27.9 ng/mL where it peaked. A transthoracic echocardiogram (TTE) showed a left ventricular ejection fraction (LVEF) of 61%. However, it did show akinesis of the mid-inferolateral walls and features consistent with a pseudonormal left ventricular filling pattern, concomitant abnormal relaxation, and increased filling pressure consistent with a Grade II diastolic dysfunction. Echocardiogram images displaying diastolic dysfunction are shown in Figure 3.

Additional laboratory workup showed normal thyroid-stimulating hormone (TSH), low-density lipoprotein (LDL) of 110, and a hemoglobin A1C of 5.3%. His calculated atherosclerotic cardiovascular disease (ASCVD) 10-year risk score was only 5%. The patient was initiated on dual anti-platelet therapy (DAPT) for an anticipated 12 months and discharged home. The patient continues to follow-up in the cardiology clinic and continues to smoke; however, he has reduced the amount of marijuana use.

## Discussion

Herein, we describe the case of a myocardial infarction that occurred in the setting of marijuana use in a young male with no significant past medical history. Marijuana generally exerts adverse events on the neurological system and does not traditionally afflict the cardiovascular system. Though uncommon, marijuana has been linked to reported cases of myocardial infarction (MI) [5]. In 2001, Mittleman et al. conducted a case-crossover study involving 3,882 patients with acute MI where they questioned the patients about marijuana use up to one hour prior to MI and found that the risk for developing myocardial infarction was 4.8 times higher than average in the hour immediately after marijuana use [2]. Our patient had also been smoking marijuana just one hour prior to the onset of his MI. Incidentally, our patient had underlying coronary artery disease, which was undiagnosed as the patient (up until the presentation) was asymptomatic. In addition to the increased risk of incurring an MI as previous studies have mentioned, the risk of post-MI mortality has also been shown to be increased in marijuana users. In a study of 1,913 adults after hospitalization for myocardial infarction, Mukamal et al. found a 4.2-fold increased risk for mortality in marijuana users who reported consuming the drug more than once per week before the onset of the infarction compared with nonusers [5].

There are many theories to why the use of cannabis may precipitate adverse cardiogenic events. One common thought process is that tetrahydrocannabinol (THC), the active component of marijuana, may affect the underlying cardiac physiology. THC works by acting on cannabinoid membrane receptors CB1 and CB2. CB1 receptors are known to be found in the brain and peripheral tissues, such as cardiac muscle, liver, gastrointestinal tract, vascular endothelium, and vascular smooth muscle cells, while CB2 receptors are found mainly in immune cells and endothelial cells and are upregulated by pro-inflammatory cytokines [6]. Though CB2 receptors have been shown to have an anti-atherosclerotic effect, as was shown by Steffens et al. in his study, administration of low-dose THC was shown to decrease progression of atherosclerotic lesions in the aortic root and abdominal aorta by decreasing monocyte adhesion and infiltrating the subendothelial region via activation of CB2 receptors on these cells [6]. THC was also found to significantly decrease interferon‐γ compared to interleukin‐10, suggesting that the anti-atherosclerotic properties of cannabinoids might be due to downregulation of the TH1 immune response [7]. A study by Sugamura et al. has shown that atherosclerotic coronary artery sections from patients with unstable angina had significantly higher expression of CB1 receptors as compared to coronary artery sections from patients with stable angina [8]. Endothelial injury also seems to play a crucial role in the development of atherosclerotic lesions. Rajesh et al. demonstrated that stimulation of CB1 receptors located on the human coronary artery endothelial cells led to an increased production of reactive oxygen species, mitogen-activated protein kinase (MAPK) activation, and endothelial cell injury and that these effects were inhibited by CB1 receptor blockade [9]. In their study, they also showed that CB2 agonism led to a decrease in the expression of inflammatory cytokines, thus decreasing endothelial injury.

Another possible mechanism of myocardial infarction is known as the coronary slow flow phenomenon (CSFP). It is an angiographic finding defined as the slow movement of contrast throughout the coronary lumen in the absence of epicardial coronary stenosis [10]. The major risk factors for CSFP include diabetes, hypertension, hyperlipidemia, and smoking. The majority of cannabis abusers do not have the risk factors for coronary heart disease (CHD), although their angiographic examinations show prominent atherosclerosis. This demonstrates that cannabis can contribute to atherosclerosis advancement, endothelial dysfunction, and the development of coronary slow flow (CSF). A mechanism by which marijuana induced-spasm eventuates in endothelial denudation at the site of a stenotic or non-stenotic susceptible atherosclerotic plaque in reaction to hemodynamic stressors has been suggested by Yurtdaş and Aydin [11]

Evidence also exists of possible coronary artery vasospasm in response to an allergic stimulus. Such a phenomenon is referred to as the Kounis syndrome and has been described in response to antibiotics, as well as other stimuli [12]. The Type II variant of Kounis syndrome occurs in patients with preexisting atherosclerotic plaques that erode and rupture due to allergen-induced inflammatory response, leading to an acute coronary syndrome. As discussed above, there are multiple known mechanisms by which marijuana can lead to myocardial ischemia; as more is known, we hope that it will make the public more aware of the implications of cannabis use.

In summary, we have presented a very interesting case of marijuana-induced NSTEMI in a 42-year-old man who had smoked marijuana an hour prior to the onset of symptoms.

## Conclusions

Marijuana or cannabis utilization is not often implicated in cardiovascular side effects. There have been a few incidental reports of the myocardial infarction directly after its utilization. Possible theories include the induction of pro-inflammatory cytokines, coronary flow abnormalities, and autoimmune response. Clinicians should be aware of the physiologic effects of marijuana and the possible role in acute coronary syndromes, including myocardial infarction. 
